# Throughput and Energy Efficiency of a Cooperative Hybrid ARQ Protocol for Underwater Acoustic Sensor Networks

**DOI:** 10.3390/s131115385

**Published:** 2013-11-08

**Authors:** Arindam Ghosh, Jae-Won Lee, Ho-Shin Cho

**Affiliations:** 1 Department of Electronics and Communication Engineering, NIT Durgapur, Durgapur 713209, India; E-Mail: arindam.gm@gmail.com; 2 School of Electronics Engineering, Kyungpook National University, Daegu 702-701, Korea; E-Mail: jwlee@ee.knu.ac.kr

**Keywords:** automatic repeat request, cooperative transmission, underwater acoustic sensor network, RCPC codes

## Abstract

Due to its efficiency, reliability and better channel and resource utilization, cooperative transmission technologies have been attractive options in underwater as well as terrestrial sensor networks. Their performance can be further improved if merged with forward error correction (FEC) techniques. In this paper, we propose and analyze a retransmission protocol named Cooperative-Hybrid Automatic Repeat reQuest (C-HARQ) for underwater acoustic sensor networks, which exploits both the reliability of cooperative ARQ (CARQ) and the efficiency of incremental redundancy-hybrid ARQ (IR-HARQ) using rate-compatible punctured convolution (RCPC) codes. Extensive Monte Carlo simulations are performed to investigate the performance of the protocol, in terms of both throughput and energy efficiency. The results clearly reveal the enhancement in performance achieved by the C-HARQ protocol, which outperforms both CARQ and conventional stop and wait ARQ (S&W ARQ). Further, using computer simulations, optimum values of various network parameters are estimated so as to extract the best performance out of the C-HARQ protocol.

## 1. Introduction

Underwater acoustic sensor networks (UASNs) are attracting huge interest due to their wide variety of applications, such as environmental monitoring, resource investigation, detection of phenomena related to natural disasters, *etc.* Naturally, all these mission-critical applications highly require reliable data transmission techniques.

To design a feasible transmission scheme, it is important to take into account the harsh and limited conditions of the underwater environment. Radio signals suffer from severe path losses in underwater scenarios, thus acoustic signals are typically employed. However, underwater acoustic links also suffer from path losses, time varying multi-path fading, motion-induced Doppler spread and aquatic noise. As a result, the underwater acoustic link often offers a high bit error rate (BER). To establish reliable communications under such poor channel conditions, an efficient retransmission scheme is needed. In general, Automatic Repeat reQuest (ARQ) schemes have been used to achieve high reliability in data transmission. Especially, in underwater acoustic sensor networks, the S&W ARQ protocol has been employed as the only way of retransmission due to the half duplexing mode of underwater acoustic modems [1,2], but high BER of the acoustic channel along with long propagation delays are making it more challenging to achieve high throughput efficiency with the conventional S&W ARQ. In recent years, several modifications in S&W ARQ have been used to improve its efficiency. Cooperation-based ARQ is one of these alternatives which utilizes the communication channels of neighbor nodes to achieve a form of cooperative diversity that can provide significant performance improvement. Particularly for UASNs, CARQ has been studied to achieve throughput enhancements [3], among other benefits [4–7].

For terrestrial wireless sensor networks (WSNs) too, cooperation-based ARQ strategies have been studied and proved very efficient [8,9]. As evident from the past literature, ARQ techniques have always benefitted from the utilization of FEC codes for improving their performance; obviously, the cooperation-based ARQ schemes are also eligible to employ them. For terrestrial wireless networks, the expected enhancement in performance when a cooperation-based ARQ is combined with the error correction technique of IR-HARQ, to form a cooperative hybrid ARQ, has also been verified [10–12].

Naturally, this concept of cooperative hybrid ARQ can be applied to UASNs as well, so as to extract the best performance, at least from the link layer, under the constraints of the harsh underwater environment. However, the performance results of cooperative hybrid ARQs obtained for terrestrial sensor networks cannot be directly applied to underwater sensor networks, because of the significant distinctions in BER and propagation delay (sound speed = 1,500 m/s, speed of radio signals = 3 × 10^8^ m/s). Especially for underwater environments, the one-by-one transmission of the redundancies of IR-HARQ might accumulate enough long delays to nullify any performance gains achieved by error-correction coding. Thus, in order to fully extract the performance gains of C-HARQ, the protocol needs to be designed and studied exclusively for underwater scenarios. To the best of our knowledge, a cooperative hybrid ARQ (C-HARQ) protocol for UASNs has not yet been designed and investigated; hence this paper presents a C-HARQ protocol which employ RCPC codes to implement IR-HARQ along with cooperation features.

Moreover, sensor networks have large numbers of nodes located in close proximity with each other, which makes it important to consider the effect of multiple neighbor nodes on throughput and energy efficiency when designing any cooperative protocol for UASNs. However, the previous analysis considers only single relay assisted cooperation [10–12]. In this paper, C-HARQ has been designed keeping in mind the presence of multiple sensor nodes in the neighborhood, which could play a significant role in improving the throughput performance.

Further, the underwater sensor nodes have limited energy sources (batteries) which cannot be easily replenished; that makes energy consumption a major issue when designing any sensor network protocol. Even high throughput protocols must be energy efficient in order to extend the network lifetime for long lasting sensor applications. Thus, energy efficiency has been used as one of the metrics for performance evaluation, along with throughput efficiency. Additionally, we compare the energy consumption burden that is carried by the neighbor nodes in providing cooperation for CARQ and C-HARQ protocols. It reveals that C-HARQ transfers less burden on the neighbor nodes compared to CARQ, while still fully utilizing them for cooperation.

Some earlier work on combination of ARQ and FEC has been done for underwater scenarios [13,14]. A hop-by-hop and block-by-block technique of transferring encoded packets has been adopted [13], but lacks the utilization of cooperative transmission. Relay-assisted HARQ has been studied [14], which primarily focused on the implementation of Luby Transform (LT) codes in IR-HARQ, not on the energy consumption analysis, or the effect of multiple relays on throughput and energy expenditure.

In this paper, we propose and study the C-HARQ protocol of error control for underwater scenarios using RCPC codes to maximize throughput and energy efficiency. The main contributions of this paper can be summarized as follows:

1. Achievement of better throughput performance by C-HARQ, compared to CARQ and S&W ARQ under similar network conditions.
2. Optimum data packet size for C-HARQ.
3. Optimum number of FEC packets for C-HARQ.
4. Maximum achievable throughput performance of C-HARQ.
5. Performance comparison in terms of energy consumption.
6. Performance analysis of C-HARQ against varying BER.

The rest of the paper is organized as follows: Section 2 presents the preliminaries to the C-HARQ protocol. Section 3 presents C-HARQ protocol design in detail, including the network model and the packet format. The performance metrics used to compare the various protocols are defined and expressed in Section 4. Section 5 presents the simulation results and their discussions in detail. Finally, the concluding remarks are presented in Section 6.

## 2. Preliminaries to the C-HARQ Protocol

The basic idea behind C-HARQ is to merge cooperative ARQ with a hybrid ARQ technique, so as to utilize the benefits of both the schemes. While CARQ can provide a form of spatial diversity, hybrid ARQ introduces error correction capabilities, thus presenting the possibility of achieving both high throughput and efficient performance. Before going into the details of C-HARQ, a brief discussion of hybrid ARQ and CARQ is presented in the following subsections.

### 2.1. Incremental Redundancy-HARQ and RCPC Codes

For any wireless network, when a basic ARQ scheme is combined with forward error-correcting coding, the resulting error-control scheme is called Hybrid ARQ (HARQ). In HARQ, the original data is encoded with a FEC code, and the redundant FEC bits are either immediately transmitted along with the data or on request from the destination when it detects errors. Based on these two different techniques, two types of HARQ are defined: Type-I and Type-II. In Type-I HARQ, the source always transmits/retransmits the entire code (consisting of data bits and redundant FEC bits) to the destination, whereas in Type-II HARQ (also known as Incremental Redundancy-HARQ), only part of the data is transmitted at first, and the FEC part (which may be further divided into smaller FEC packets) is sent later to the destination, when required. The destination then combines the erroneous data packets with these transmitted redundancies to attempt forward error correction. Generally, Type-I HARQ suffers from capacity losses under good channel conditions because of the extra redundancy introduced, which may be twice the size of data in some FEC codes, whereas, IR-HARQ does not suffer from such a loss, as it expends channel capacity only when needed; it also performs with good sensitivity under poor channel conditions because of the coding gain. Thus, in this paper, IR-HARQ is selected as the HARQ technique, to be combined with CARQ, primarily because of its on-demand method of forward error correction.

To implement IR-HARQ, the most commonly used error-correcting codes are the rate-compatible punctured convolution (RCPC) codes [15]. They employ a convolution encoder equipped with predefined puncturing patterns to generate redundancies of IR-HARQ. At first a suitable convolution encoder is used to encode *d-bit* data into *m-bit* codewords called the Mother code (code rate = *d*/*m*). Then, by using a suitable puncturing pattern, some bits in the Mother code are deleted, to be left with a punctured codeword of higher code rate. The punctured mother code forms the message for original transmission and the deleted bits are enclosed into multiple packets to form the FEC packets, which are sent later. The probability of error correction increases when the erroneous message is decoded along with the following FEC packets. The detailed description of the packet formation technique, using convolution coding and puncturing, is presented in Section 3.

### 2.2. Cooperative ARQ

CARQ is basically a stop-and-wait mode of error control that exploits a form of spatial diversity provided by the nodes in the neighborhood of the source and destination. This spatial diversity is achieved by employing the neighbor nodes to work in cooperation with the source node during the retransmission process. The possibility of cooperation arises from the fact that transmission over wireless channels is a broadcast in nature and thus allows the neighbor nodes to overhear the data transmitted by the source. This phenomenon enables the neighbor nodes to act as alternate sources for the current data. In case of failure in data transmission from the main source node, the destination calls for cooperation from these alternate sources to transmit their overheard data. The main advantage of this method lies with the possibility of finding multiple, and relatively better, communication channels of the neighbor nodes which significantly increases the chance of successful retransmission. Thus, this cooperation based operation boosts the throughput performance of an ordinary S&W ARQ to a much higher level [3]. The performance of the simple CARQ can be further improved if the error-correcting codes could come into play. To realize that, C-HARQ protocol is designed in the following section.

## 3. Proposed C-HARQ Protocol Design

The merger of CARQ and IR-HARQ can be achieved primarily in two ways: (1) using the neighbor nodes to transmit data as well as error correction information; (2) using the neighbor nodes to transmit only the error correction information. In case 1, the neighbor nodes simply act as an alternate source of data as well as error correction bits; consuming the same amount of energy as the source, thus losing a significant part of its own energy resource in delivering others' data. Case 2, on other hand, tries to transfer as less a burden as possible to the neighbor nodes while still fully utilizing their cooperation. This is accomplished by asking the neighbor nodes to transmit only the error correction redundancies, called the FEC packets, when needed, to the destination, while it remains the source responsible for the data part. The smaller size of the FEC packets causes comparatively less energy consumption. Moreover, an FEC packet from a neighbor node can still be utilized even if it gets corrupted in some bits, whereas even a single error in data packet will result in wastage of the resources consumed in transmitting that packet. Thus, for the C-HARQ protocol we prefer the second option, where the neighbor nodes are burdened of transmitting only the smaller FEC packets that carry the information for correcting errors, thus providing the opportunity of boosting throughput performance with better energy efficiency. The system model used to implement the C-HARQ protocol along with the detailed description of the cooperation mechanism employed to transfer the FEC information to the destination is presented as follows.

### 3.1. Network Scenario

**Figure 1.**
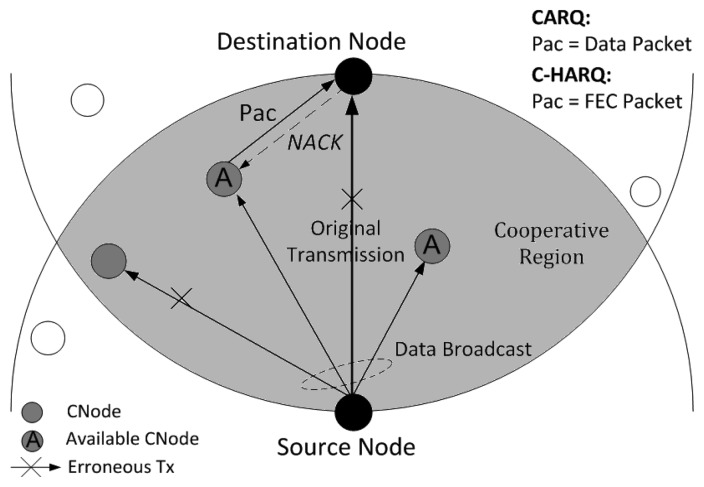
Network Configuration.

As this work primarily focuses on the ARQ aspect, not on medium access control (MAC) issues, we have assumed that all the media access issues have been resolved so that the underwater sensor network under consideration is only a single-hop case between a source and a destination with some neighbour nodes in the surroundings. All the nodes remain static at their respective positions. The network scenario is depicted in Figure 1.

The overlapped area between two circles having a same radius of the source-to-destination distance, each centered at source and destination is called the cooperative region. Any neighbour node within this region can overhear any transmission from the source and destination and, hence, has the potential to provide cooperation; thus these nodes are hereafter referred to as cooperative nodes (*CNodes*).

### 3.2. The Cooperation Mechanism

The main cooperation mechanism of C-HARQ protocol is basically similar to that of CARQ. A success in the original data transmission is acknowledged, by the destination, by sending a positive acknowledgement (*ACK*) signal to the source; whereas on reception of erroneous data, destination opts for a cooperation process instead of immediately sending a negative acknowledgement (*NACK*) signal. Meanwhile, some (or all) of the *CNodes* becomes available for cooperation (holding either a data packet, for CARQ, or FEC packets, for C-HARQ) depending on whether they succeeded or failed in correctly overhearing the broadcasted data. When the destination calls for cooperation, these available *CNodes* let the destination know about their availability by a proper signaling mechanism (described later in Section 3.4). The destination, then, asks the available *CNodes*, one-by-one, to transmit the packets they hold until the retransmission/recovery is successful. A successful recovery of original data from the cooperation process is conveyed to the source and the *CNodes* by transmitting an *ACK*. However, if the cooperation process fails too, a *NACK* is transmitted to ask the source to retransmit the data, which starts the whole process from the beginning.

### 3.3. Packet Generation

The system consists of mainly three types of packets: data packets (main message packets), FEC packets and signalling packets. The message packet and FEC packets are generated using a convolution encoder with puncturing enabled. The punctured mother code at the output of the encoder forms the main message packet while the punctured bits form the FEC packets. The convolution encoder is configured in such a way that the punctured mother code produced is exactly the same as the original data that was fed to the input of the encoder. Thus, the main message packet contains only data bits and is, hereafter, referred to simply as the data packet. This allows the source to be exempted from the burden of going through the encoding process to generate the main message packet which is actually the data part only. The other punctured outputs of the encoder form the FEC packets.

**Figure 2.**
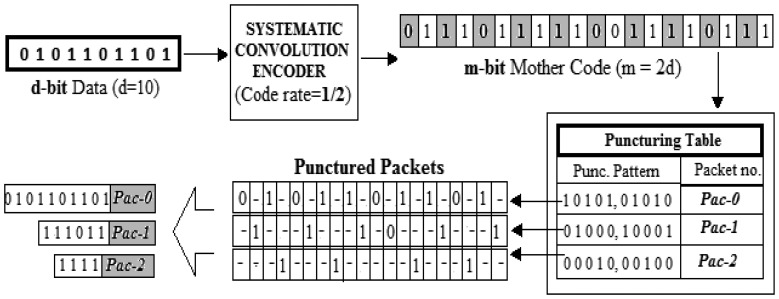
Packet Generation.

The convolution encoder thus used is a *systematic convolution encoder* [16]; a code is systematic if the input data bits are embedded in the output mother code. Figure 2 explains the coding and the puncturing process with an example. At first a 10-bit data is encoded into a 20-bit mother code (code rate = 1/2) with data bits (highlighted bits) and FEC bits occupying the odd and even positions, respectively. Next, the mother code is punctured using some predefined puncturing patterns to get multiple packets at the output. By using the pattern [1010101010], the redundant FEC bits are deleted from mother code (positions with “−”) to get the payload of the data packet *i.e.*, *Pac*-0. Thus, *Pac*-0 contains only the data bits and therefore, from hereafter, refers to the original data packet in the system. Similarly, utilising the other puncturing patterns, FEC bits are recovered from the mother code to be the payload of the FEC packets; represented by *Pac*-1, *Pac*-2…*Pac-N_mx_*. In the above example, *N_mx_* is set to 2.

**Figure 3.**
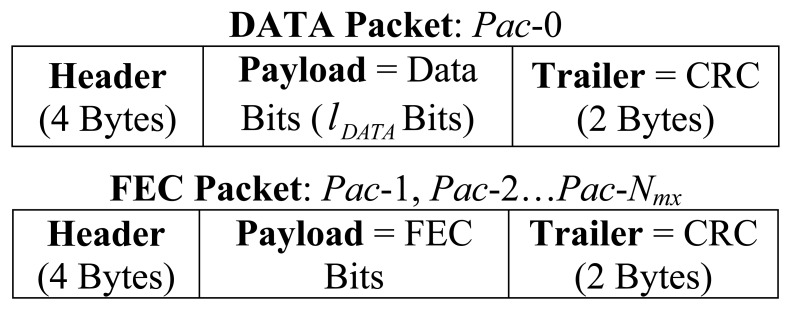
Final Link Layer Packet Format.

To these raw packets, a four byte header and a two byte trailer are added to form the final link layer packets, shown in Figure 3. The header field consists of packet ID (1 Byte), source ID (1 Byte), destination ID (1 Byte), and packet sequence number (1 Byte). The trailer is composed of 16-bit Cyclic Redundancy Check (CRC) to facilitate error detection.

### 3.4. Scheme Description

We now proceed to describe the C-HARQ protocol of error-control for data transmission from a particular source to a destination. The scheme works in the following subsequent phases:

1. Transmission of *Pac*-0 by Source.
2. Discovery of available Cooperative nodes.
3. Cooperation process.
4. Transmission of *ACK*/*NACK* by Destination.

Phases 2 and 3 come into play only when an error is detected at the destination. As mentioned earlier, the source is not involved in any convolution encoding process. The source is only concerned with sending/resending the current or the next data packet, from its packet buffer, depending on the type of acknowledgement received from the destination. Encoding process is required only at the *CNodes* to generate the FEC packets using the overheard *Pac*-0.

The source starts Phase1 by transmitting the *Pac*-0 that is present at the top of its packet buffer, to the destination. This transmission, which is a broadcast in nature, is also overheard by the *CNodes*. The *CNodes* individually compute CRC to check for errors in the overheard *Pac*-0. Those *CNodes* which correctly receive *Pac*-0, encode it into *Pac*-1, *Pac*-2…*Pac-N_mx_*, and saves them at the top of their individual packet buffer, to become “*available*” for cooperation. Other *CNodes* discard the erroneous overhearing and remain silent.

Meanwhile, at the destination end, error detection is applied to the received *Pac*-0 and steps are taken accordingly:

No Errorin recevied Pac‐0:

Phase 2 and 3 are skipped and an *ACK* is immediately sent to the source by destination. Upon overhearing *ACK*, the *CNodes* drops the current FEC packets from the top of their packet buffer:

Errordetected in recevied Pac‐0:

Destination saves the erroneous *Pac*-0 in its packet buffer as *saved_Pac*-0 and enters Phase 2 by broadcasting a “*Request for Cooperation*” (*C_Req_*) signal. Prior to the broadcast, the destination initializes enough memory space in its packet buffer for the upcoming FEC packets meant for error correction. On overhearing the *C_Req_* signal, the source continues to remain in “*idle*” mode waiting for an *ACK or NACK* signal to respond to. By doing so it temporarily leaves the responsibility of recovering *Pac*-0 to the cooperation process. In our system model, we have assumed that a node operates in *idle*-mode when it is involved in none of the following activities: transmitting, receiving or convolution decoding.

Meanwhile, the available-*CNodes* respond to *C_Req_* by individually transmitting the “*Availability for Cooperation*” (*C_Avl_*) signals to the destination. To make the collisions of *C_Avl_* less likely, a proper media access protocol (like a Random Back-Off algorithm) can be used here. However, for our performance analysis, this probability of collision is neglected due to the very small size of *C_Avl_* and location of *CNodes* at a random distance from the destination. The destination, after transmitting *C_Req_*, waits for a maximum of “*Tc*” amount of time to allow all the availability signals to reach the destination before the commencement of the cooperation process. The value of “*Tc*” is set to twice the source-to-destination propagation delay so that the *C_Avl_* signals, even from the farthest *CNodes* (located at a distance of source-to-destination distance from both the source and the destination), have enough time to reach the destination. Upon expiration of this waiting time, the destination saves all the received *C_Avl_* signals in a list, called the “*Look-Up*” list. This list is mainly used to select or look for the current or the next available-*CNode* during the cooperation process. Prior to the cooperation process, the destination applies a predefined selection criterion to sort the list in a particular order. For this purpose, the *C_Avl_* signal can be designed to contain multiple fields for holding information related to the available-*CNodes*, such as, distance from destination, remaining level of energy, channel conditions *etc.*

An empty *Look-Up* list indicates that none of the *CNodes* have successfully overheard *Pac*-0 and the process goes into Phase 4, skipping the cooperation process (Phase 3), by transmitting a *NACK*. The source then starts again from Phase 1 by retransmitting *Pac*-0 to the destination. Otherwise, if even a single *CNode* is available, the cooperation process is deployed as follows:

### Cooperation Process

The first step of the process is to select a *CNode* from the *Look-Up* list, for the destination to contact first. The simplest criteria would be to select them on the basis of their closeness to the destination, which can be realized by allowing the *C_Avl_* signal to contain certain timestamp information which the destination can use to calculate the round-trip delay between itself and that *CNode*. The *CNode* with the least round-trip delay can be chosen to be the best one. However, if this option is not available, a simple First-Come-First-Serve (FCFS) selection procedure can be used, where the *CNodes* are selected in the order of their *C_Avl_* arrival. The former is assumed to be the selection criteria for our analysis.

Next, the process of contacting a *CNode* basically involves transmission of a *NACK*_j_ to the *i* th available-*CNode* (*CNode*-i), asking for the FEC packet *Pac*-j. Following this mechanism the destination sends a *NACK*-1 to the closest *CNode*, which can be referred to as *CNode*-1. Upon receiving *NACK*-1, *CNode*-1 transmits *Pac*-1 to the destination. Destination saves the received *Pac*-1 as *saved_Pac*-1 then checks for errors in it by computing its own CRC. Here, the destination needs to maintain a special “*status*” vector so as to keep track of the status of the saved FEC packets; where, *status*=1 means the received FEC packet is error-free and doesn't require any retransmission, while *status*=0 means the *saved_Pac*-1 has some errors, and thus may require retransmission. Then, the destination tries to recover the original data by combining the *saved_Pac*-0 and *saved_Pac*-1 and then feeding it to the Viterbi decoder [17]. If the recovery is successful, an *ACK* is sent to the source and the cooperation process terminates, otherwise, *NACK*-2 is sent to *CNode*-1 for the next FEC packet, *Pac*-2. On receiving *Pac*-2, destination saves it as *saved_Pac*-2 with its status set accordingly, and then utilizes it in the next Viterbi decoding attempt along with the previously saved packets. This process of FEC packet reception and Viterbi decoding continues in association with *CNode*-1 till the original data gets recovered or the transmission of the last FEC packet (*Pac-N_mx_*) from *CNode*-1 gets completed. In case the later happens and the error still persists, it is because of the either of the two possible cases: (1) none or only few of the total required correct FEC packets (error-free versions) are available, thus unable to fully utilize the error correction capability or (2) all the saved-FEC packets are correct but the count of errors in *saved_Pac*-0 is beyond the error correction capability of the convolution code. In the first case, the destination approaches the next closest available-*CNode*, *CNode*-2, for further cooperation, where, before sending a *NACK*, destination first checks the *status* vector to identify all the incorrect FEC packets and sets the value of the *NACK* accordingly. For example, if retransmission of *Pac*-j is required, the value of *NACK* is set to “j”. On reception of *NACK*_j_, *CNode*-2 finds the *Pac*-j from its buffer and transmits it to the destination. Destination overwrites the *saved_Pac*-j with the freshly received *Pac*-j; performs error detection and accordingly updates its *status*. As usual, a Viterbi decoding is carried out using all the packets saved in the buffer including the new one. A successful recovery results in termination of the cooperation by sending an *ACK* to the source, otherwise, the attempts of error correction continues for other FEC packets and with other available-*CNodes*.

The second case, where all the FEC packets have been correctly saved (*i.e.*, *status* = 1 for all FEC packets), implies that no more cooperation is required. Thus to terminate the current cooperation process, and to ask the source for a new copy of *Pac*-0 (so as to replace the *saved_Pac*-0), a *NACK* is sent to the source. Upon receiving the *NACK*, the source comes out of the idle mode and transmits a fresh copy of *Pac*-0, which is then utilized by the destination in the following error correction attempt. If the error correction fails again, a *NACK* is sent to the source again. Here, the destination requests for no further cooperation as all the FEC packets are already available. Now, the destination and the source continue to work in this S&W ARQ manner, with the error correction information available at the destination, until the data is successfully recovered.

In another special case where the last available-*CNode* has been contacted but some of the saved FEC packets still require retransmission, a *NACK* is sent to the source, so as to eventually start the next cooperation process for acquiring the remaining FEC packets. On hearing the *NACK*, source simply retransmits *Pac*-0, with which the destination first attempts Viterbi decoding utilizing the previously saved FEC packets; if the data is recovered successfully an *ACK* is sent, otherwise the next cooperation process is initialized from the stage of *C_Req_* transmission so as to obtain the remaining FEC packets.

## 4. Performance Analysis

In this section we define the expressions of the performance metrics, *i.e.*, throughput efficiency and energy efficiency, which is used to analyse the performance of the C-HARQ protocol against the CARQ and S&W ARQ protocols.

### 4.1. Throughput Efficiency

The throughput efficiency of an error control scheme is defined as the ratio of the transmission time of the data packet to the total time taken in successfully delivering the packet. The throughput efficiency of our system is thus expressed as:

(1) $$\varepsilon_{\text{tpt}}=\frac{T_{t}\cdot l_{\text{DATA}}}{T_{t}\cdot l_{\text{Pac}-0}+2T_{p}+T_{co-op}}$$

where *T_t_* is the transmission time of a single bit, *T_p_* is the source-to-destination propagation delay and *l_DATA_* is the data payload length of *Pac*-0. Time duration 2*T_p_* accounts for the round trip delay caused by the very first original transmission of the data packet and the final reception of the *ACK* at source. In between these two events, if the cooperation process comes into play the extra delay incurred is accounted by *T_CO-OP_*.

### 4.2. Energy Efficiency

Before defining the energy efficiency metric for the cooperative protocols, we consider the simple case of single hop S&W ARQ communication between the source and the destination. The total energy consumed in the network for a single attempt of data packet delivery by source (that results in an *ACK* or *NACK* response from the destination) is given by:

(2) $$E_{\text{ARQ}}=E_{\text{ARQ}}^{S}+E_{\text{ARQ}}^{D}+E_{\text{ARQ}}^{CN}$$

where $E_{\text{ARQ}}^{S}$, $E_{\text{ARQ}}^{D}$ and $E_{\text{ARQ}}^{CN}$ are the energies consumed at the sender, the destination and the neighbour nodes (*i.e.*, cooperative nodes for the cooperative protocols) respectively, and are further expressed as:

(3a) $$E_{\text{ARQ}}^{S}=E_{Tx}^{\text{DATA}}+E_{Rx}^{\text{ACK}/N\text{ACK}}+E_{\text{Idle}}^{S}$$

(3b) $$E_{\text{ARQ}}^{D}=E_{Rx}^{\text{DATA}}+E_{Tx}^{\text{ACK}/N\text{ACK}}+E_{\text{Idle}}^{D}$$

(3c) $$E_{\text{ARQ}}^{CN}=\sum_{i=1}^{\text{nCN}}E_{\text{Idle}}^{i}$$

where, for any network packet “*Pac*”, *Pac* ϵ {*DATA*, *FEC*, *ACK*, *NACK*, *C_Req_*, *C_Avl_*} and length *l_Pac_*, $E_{Tx}^{\text{Pac}}$ and $E_{Rx}^{\text{Pac}}$ are the energies consumed in transmitting and receiving that packet, respectively. *E_Idle_* is the energy consumed by a node when operating in the idle mode. *nCN* denotes the number of *CNodes* that are present in the neighborhood of the source and destination (*i.e.*, in the cooperative region). For S&W ARQ the *CNodes*, obviously, do not interfere, and hence only their idle mode energy consumption is considered. The above mentioned energy consumptions can be further expressed as:

(4a) $$E_{Tx}^{\text{Pac}}=P_{Tx}l_{\text{Pac}}T_{t}$$

(4b) $$E_{Rx}^{\text{Pac}}=P_{Rx}l_{\text{Pac}}T_{t}$$

(4c) $$E_{\text{Idle}}=P_{\text{Idle}}t_{\text{Idle}}$$

where, *P_Tx_*, *P_Rx_* and *P_Idle_* are the transmission power, reception power and idle mode operating power, respectively. *t_Idle_* is the total amount of time for which a node operates in idle mode, during the entire process.

Using the same methodology as S&W ARQ, the energy consumption for both CARQ and C-HARQ are obtained next; where, we present the energy consumption model, employed during the computer simulation of the cooperative protocols, by expressing the energy efficiency equations for a very simple example case that considers a major assumption. It is to be noted that the computer simulations are fully fair, without any of the assumptions; whereas the example case is considered, in the following part, only to give an insight into the energy consumption model.

#### 4.2.1. Cooperative ARQ

The cooperative ARQ protocol considered in this paper, for comparison with C-HARQ, is basically the same as the previous one [3], but with the addition of *CNode* discovery process using *C_Avl_* and *C_Req_* signals; whereas previously it was assumed that the destination has a prior knowledge of all the *CNodes*. Rest of the process of one-by-one cooperation of *CNodes* starting from the closest *CNode* first, is same.

For the example case, let the source transmit a data packet *sTx* times in total, invoking a total of *sTx* cooperation processes, until the data packet is successfully delivered to the destination; during the final, *sTx*-th, cooperation process, *f* numbers of *CNodes* are contacted before the success. The cooperative region contains a maximum *nCN* number of potential *CNodes*, and the main assumption here is that all of them become available in the very first cooperation process. Then, the total energy consumption of the entire CARQ process can be calculated as the sum of the energies consumed at every node, which can be expressed as:

(5) $$E_{\text{CARQ}}=E_{\text{CARQ}}^{D}+E_{\text{CARQ}}^{S}+E_{\text{CARQ}}^{CN}$$

where $E_{\text{CARQ}}^{D}$, $E_{\text{CARQ}}^{S}$ and $E_{\text{CARQ}}^{CN}$ are the energies consumed respectively at source, destination and all the *CNodes* combined. The total energy consumption of the destination node can be given by:

(6) ECARQ|sTx=rf=pD={r+(r−1)nCNA+p}ERxDATA+ETxACK+(r−1)ETxNACKS+{(r−1)nCNA+p}ETxNACKCN+rETxCReq+nCNAERxCAvl+EIdleD

where, *nCN_A_* denotes the number of *CNodes* that are available for cooperation, and considering that all *CNodes* become available, *nCN_A_* = *nCN*. Next, we define Pr(*f* = *p* | *sTx* = *r*) to be the probability that for a successful delivery it takes *r* data packet transmissions from the source, and the final delivery coming only after *p CNodes* have been contacted during the *r*-th cooperation attempt, with *r* ∈ {1,2,3 …∞} and *p* ∈ {0,1,2 …*nCN_A_*}, such that:

(7) $$E_{\text{CARQ}}^{D}=\sum_{r=1}^{\infty }\sum_{p=0}^{nCN_{A}}\left\{E_{\text{CARQ}|\begin{array}{l} \text{sTx}=r\\ f=p \end{array}}^{D}\times \mathrm{Pr}(f=p|\text{sTx}=r)\right\}$$

In the illustration of Equation (6), the destination receives the data packet *r* times from the source, (*r* − 1) *nCN_A_* and *p* times from the *CNodes*, through first *r* − 1 cooperation processes and the final cooperation process respectively. Destination transmits *r* − 1 number of *NACK*_s_ (*NACK*-to-Source) in response to the first *r* − 1 unsuccessful attempts and an *ACK* for the successful, *r*-th, one. Further, (*r* – 1) *nCN_A_* + *p* numbers of *NACK_CN_* (*NACK*-to-*CNodes*) are sent by the destination to *CNodes*, during the entire cooperation process, to ask for data packet retransmission. Here, *NACK_S_* and *NACK_CN_* are basically the same negative acknowledgement signal, but used only to differentiate between a *NACK* sent to ask the source to transmit and a *NACK* sent to ask a *CNode* to transmit, respectively. Next, considering the fact that destination lacks prior knowledge of the total number of potential *CNodes* in the cooperative region, it initiates every cooperation attempt with the *CNode* discovery process (through the broadcast of *C_Req_* signal), even when in actual all the *CNodes* have become available; hence, a total of *r C_Req_* signals are transmitted by the destination. Further, during the computer simulation, the *C_Avl_* signal has been considered immune to errors thus making it sufficient for an available *CNode* to send the *C_Avl_* signal only once to the destination, even if it keeps hearing *C_Req_* signals thereafter; hence the destination receives a total of *nCN_A_* number of *C_Avl_* signals during the entire process. To reduce the time overhead caused due to this repetitive *CNode* discovery process even when not required, we present in the result section a maximum limit that can be set to the number of discovery attempts, particularly for the C-HARQ protocol, after which the destination directly goes into the cooperation, skipping the *CNode* discovery process. Next, the energy consumption at the source node can be similarly expressed as:

(8) ECARQ∣sTx=rf=pS=rETxDATA+(r−1)ERxNACKS+ERxACK+{(r−1)nCNA+p}ERxNACKCN+rERxCReq+nCNAERxCAvl+EIdleS

(9) $$E_{\text{CARQ}}^{S}=\sum_{r=1}^{\infty }\sum_{p=0}^{nCN_{A}}\left\{E_{\text{CARQ}|\begin{array}{l} \text{sTx}=r\\ f=p \end{array}}^{S}\times \mathrm{Pr}(f=p|\text{sTx}=r)\right\}$$

The source node not only receives *NACK_S_* and *ACK* signals meant for it but also overhears the *NACK_CN_*, *C_Req_* and *C_Avl_* signals that are exchanged between the destination and the *CNodes*; hence the energy consumption resulting from them is also included in Equation (8). The overhearing of these signals keeps the source informed about the ongoing cooperation activity. The data packet transmitted by a *CNode* to the destination can also be overheard by the source and rest of the *CNodes*, however it has been assumed that a node can know whether a packet is meant for it or not by processing only the initial few bits (*i.e.*, header part), and hence can immediately stop or continue receiving the rest of the packet. Especially for larger packets (*i.e.*, data and FEC packets), decoding of the packet header can be completed much before the antenna receives the rest part of the packet, as the processing takes very less time compared to the reception time. Employing this mechanism the overhearing of a *CNode*'s data packet (for CARQ) and FEC packet (for C-HARQ), by source and other *CNodes*, has been avoided during the computer simulations.

The total energy consumption of the cooperative nodes is calculated as the sum of the energies consumed by the individual cooperative nodes, which can be expressed as:

(10) ECARQ∣sTx=rf=pCN=∑i=1nCNA{ERxDATA+(r−1)ETxDATA+(r−1)ERxNACKS+ERxACK+{(r−1)nCNA+p}ERxNACKCN+rERxCReq+ETxCAvl+EIdleCNode−i}+∑1pETxDATA

(11) $$E_{\text{CARQ}}^{CN}=\sum_{r=1}^{\infty }\sum_{p=0}^{nCN_{A}}\left\{E_{\text{CARQ}|\begin{array}{l} \text{sTx}=r\\ f=p \end{array}}^{CN}\times \mathrm{Pr}(f=p|\text{sTx}=r)\right\}$$

The *CNodes* overhear the *NACK_S_* and *ACK* signals transmitted by the destination, so as to keep track of the current status of the protocol, and decide whether to retain or drop the source's overheard data packet from its packet buffer. Additionally, it can also overhear the *NACK_CN_* signals transmitted to other *CNodes*, along with the *NACK_CN_* signals meant for it. However, as mentioned earlier, a *CNode* avoids the overhearing of any other *CNode*'s data packet as it already has a copy of it.

Finally, we define the energy efficiency as the ratio of the useful energy consumed in transmitting the data packet to the total energy consumed in successfully delivering that packet. Hence, the energy efficiency of CARQ can be expressed as:

(12) $$\eta =\frac{E_{Tx}^{\text{DATA}}}{E_{\text{CARQ}}}=\frac{P_{Tx}l_{\text{DATA}}T_{t}}{E_{\text{CARQ}}^{D}+E_{\text{CARQ}}^{S}+E_{\text{CARQ}}^{CN}}$$

#### 4.2.2. Cooperative-HARQ

Again considering the same case of *sTx* times data packet (here *Pac*-0) transmissions from source, invoking a total of *sTx* cooperation processes, for the successful recovery of data at the destination, the total energy consumption of the C-HARQ protocol is given by:

(13) $$E_{\text{CHARQ}}=E_{\text{CHARQ}}^{D}+E_{\text{CHARQ}}^{S}+E_{\text{CHARQ}}^{CN}$$

At the destination, the total energy consumption can be expressed as:

(14) ECHARQ∣sTx=rD=rERxPac−0+(r−1)EdeclDATA+(r−1)ETxNACKS+ETxACK+rETxCReq+nCNAERxCAvl+EIdleD+∑i=1nCNA∑j=1Nmxnij(ETxNACK−j+ERxPac−j+EdeclDATA)

(15) $$E_{\text{CHARQ}}^{D}=\sum_{r=1}^{\infty }\left\{E_{\text{CHARQ}|\text{sTx}=r}^{D}\times \mathrm{Pr}(\text{sTx}=r)\right\}$$

Since the basic cooperation mechanism is same for C-HARQ, the *NACK_S_*, *ACK*, *C_Req_* and *C_Avl_* terms in Equation (12) are same as that in Equation (6). But, with data packets coming only from the source, the destination receives the copies only *r* times and every time a Viterbi decoding is attempted using the previously saved FEC packets, except the very first transmission; thus resulting in the second term in Equation (12), where, *l_DATA_* is the data payload size of *Pac-0* and *E_dec_* represents the energy consumed per useful bit (data bit) in a Viterbi decoding attempt. *N_mx_* is the total number of FEC packets generated by RCPC encoding and *n_ij_* represents the number of times the destination had to ask the *i-th CNode* to transmit *Pac*-j before successfully receiving and storing it. In other words, *n_ij_* represents the total number of transmissions of *Pac*-j from the *i-th CNode* during the entire process; and every FEC packet reception is accompanied by a Viterbi decoding attempt.

The source in C-HARQ is not involved in any FEC encoding or decoding process hence the working of the source is the same as that in CARQ; the only difference is the number of *NACK* to *CNodes* overheard. Again, the overhearing of a *CNode*'s FEC packet has been avoided for source as well as other *CNodes*. Thus, the energy consumption at the source node is given by:

(16) ECHARQ∣sTx=rS=rETxPac−0+(r−1)ERxNACKS+ERxACK+rERxCReq+nCNAERxCAvl+EIdleS+∑i=1nCNA∑j=1NmxnijERxNACK−j

(17) $$E_{\text{CHARQ}}^{S}=\sum_{r=1}^{\infty }\left\{E_{\text{CHARQ}|\text{sTx}=r}^{S}\times \mathrm{Pr}(\text{sTx}=r)\right\}$$

Similarly at the cooperative nodes, the total energy consumption is given by:

(18) ECHARQ∣sTx=rCN=∑i=1nCNA{ERxPac−0+(r−1)ERxNACKS+ERxACK+rERxCReq+ETxCAvl+EIdleCNode−i+∑k=1nCNA∑j=1NmxnkjERxNACK−j+∑j=1NmxnijETxPac−j}

(19) $$E_{\text{CHARQ}}^{CN}=\sum_{r=1}^{\infty }\left\{E_{\text{CHARQ}|\text{sTx}=r}^{CN}\times \mathrm{Pr}(\text{sTx}=r)\right\}$$

A *CNode* listens to all the *NACK* signals but responds only to the ones meant for it. Finally, from Equations (4a) and (13), the energy efficiency of C-HARQ can be written as:

(20) $$\eta =\frac{E_{Tx}^{\text{DATA}}}{E_{\text{CHARQ}}}=\frac{P_{Tx}l_{\text{DATA}}T_{t}}{E_{\text{CHARQ}}^{S}+E_{\text{CHARQ}}^{D}+E_{\text{CHARQ}}^{CN}}$$

Theoretical calculation of *n_ij_*, Pr(*f* = *p* | *sTx* = *r*), Pr(*sTx* = *r*) and *T_CO-OP_* are beyond the scope of this paper, hence computer simulations are used instead.

## 5. Simulation Model and Results

In this section we present the numerical results obtained from computer simulations of C-HARQ protocol for the underwater sensor network depicted in Figure 1. The entire system is modelled in a Matlab environment, where extensive Monte Carlo simulations are performed to numerically measure the performance of a protocol. The averaged values of the performance metrics obtained for C-HARQ are compared with those of CARQ and standard S&W ARQ under common network conditions. Further, the performance metrics are used to find the optimum values of the network parameters so as to optimize the overall performance.

For convolution encoding, the constraint length (*K*) of the systematic encoder is set to 5. The generator polynomial pair {37 33} is used, “37” (octal representation) being the feedback polynomial, which delivers a code rate of 1/2. At the destination a hard decision Viterbi decoder, operating in ‘*trunc*’ mode, with a trace-back length set equal to 5*K*, is used for decoding the convolution codewords along with their punctured derivatives. Out of the three operating modes of Viterbi decoder in Matlab, *i.e.*, “*cont*”, “*term*” and “*trunc*”, trunc mode is used because it incurs no delay before the appearance of first symbol at the output [16]. In general, the energy requirement of convolution encoding process is considered negligible [18,19], however the process of Viterbi decoding is quite energy-intensive. Assuming a StrongARM microprocessor (SA-1110) to be the on-board processing unit, the average energy required per useful bit (*E_dec_*) to decode a punctured convolution code (code rate = 1/2, *K* = 5) using a Viterbi decoder, has been experimentally found to be approximately 0.02 mJ [19]. The encoding and decoding energies of CRC are neglected.

The underwater acoustic channel for data transmission is basically modelled in Matlab by a binary symmetric channel (BSC), whose bit error probability value is taken from the “average BER *vs.* inter-nodal distance” graph obtained in previous woks on CARQ in UASNs [3]. The average BER is obtained for binary phase shift keying (BPSK) modulation under Rayleigh channel fading, where the signal-to-noise ratio (SNR) is primarily affected by frequency and distance dependence attenuation, and ambient noise modelled by turbulence, shipping, waves and thermal noise. For the source-to-destination link of length 1,000 m the average BER is found to be around 2.5 × 10^−3^; for the cooperative links varying from 100 m to 1,000 m the BER is found to vary in the range of 2 × 10^−5^ to 2.5 × 10^−3^. For the sensor nodes, the on-board acoustic modem is considered to be the LinkQuest UWM2000 acoustic modem [2]. The relevant specifications of the modem, along with some network parameter values, are given in Table 1.

The main assumptions or relaxations taken into account for the simulation purpose are:

1. Control signals and Header/Trailer part of a packet are immune from errors.
2. Collision-free transmission of *C_Avl_* signals.
3. No limit to source's retransmission attempts.
4. 100% error detection capability of CRC.
5. Negligible processing time compared to the long propagation delays.

To be able to apply the performance results of this protocol to any general network configuration, and not to any fixed network topology, the location of the *CNodes* are changed at the beginning of every simulation run, by randomly redistributing them in the cooperative region; the source and destination remain fixed at their positions. Further, to obtain averaged and confident values, the simulations are executed over enough number of runs (100–150), with each run delivering 1,000–1,500 data packets from source to destination, so that the final performance metrics plots are definite and smooth. Moreover, to validate the accuracy and precision of the obtained results 95% confidence interval is also shown, but only in one plot (Figure 5) due to lack of space in other figures. Nonetheless, the confidence level of the 95% confidence interval applies to each and every plot. Finally, the obtained results and their discussions are as follows.

### 5.1. Optimum Data Payload Size

**Figure 4.**
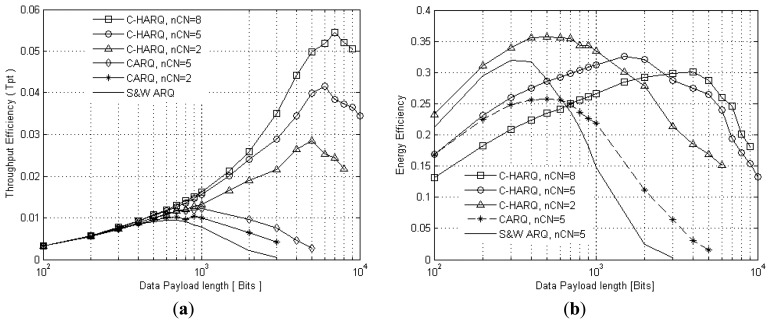
(**a**) Throughput efficiency *vs.* data payload length; (**b**) Energy efficiency *vs.* data payload length.

Figure 4a presents the throughput efficiency as a function of data payload size for the C-HARQ, CARQ, and standard S&W ARQ protocols. It clearly shows that the error correction capabilities of C-HARQ boost the throughput efficiency of CARQ to a much higher level, thus outperforming both CARQ and S&W ARQ by a big margin. Further, considering a wide range of applications of the sensor networks resulting in variation in the number of neighbour nodes, and to estimate an overall average value of the optimum data payload size, the throughput efficiency of C-HARQ is plotted for *nCN* = 2, 5 and 8. The payload size which achieves the maximum throughput efficiency is found to be in the range of 5,000–7,000 bits for *nCN* = 2 to 8. Thus, for further analysis of C-HARQ we set the optimum payload size to 6,000 bits, which is far greater than that of CARQ (1,000 bits) and S&W ARQ (600 bits); this allows more amounts of data transfer on a single successful attempt.

From the point of view of energy, the three protocols are first tested with the number of neighbour nodes set to 5; their energy efficiency is plotted in Figure 4b. Additionally, the energy efficiency of C-HARQ for *nCN* = 2 and 8 is also presented. The plot reveals that C-HARQ outperforms the simple CARQ protocol over the entire range of payload length. When compared to S&W ARQ, C-HARQ delivers more energy efficiency especially for larger data payloads (≥600 bits), but for smaller payloads (≤500 bits) S&W ARQ is found to be more energy conserving than the cooperative protocols, the reason being the additional energy consumption of the *CNodes* due to their inherent property of listening to the original data transmission even when not required, which is the case with smaller payloads where the packet error rate (PER) is low. In case of conventional S&W ARQ, it has been assumed that the *CNodes* know about the on-going communication and hence remain in idle mode for the entire duration, adding only the idle mode energy expenditure to the total consumption.

Further, C-HARQ with *nCN* = 2 proves to have better energy efficiency than *nCN* = 5 and 8, for payload of smaller size but for larger payload size *nCN* = 5 and 8 have the better efficiency. This is again because of the additional energy consumption of the *CNodes* in overhearing the original transmission when their cooperation is not required. Whereas, for larger payload size the PER is high and *CNodes* are required to play their role in improving the performance, thus overshadowing their overhearing energy expenditure. From the aspect of energy efficiency, it is difficult to assign a common optimum payload size for C-HARQ for all types of network configurations, as the optimum size is found to vary in the range of 600 bits (*nCN* = 2) to 4,000 bits (*nCN* = 8). Thus we use the optimum payload setting obtained from throughput efficiency analysis.

### 5.2. Optimum Number of FEC Packets, Nmx

**Figure 5.**
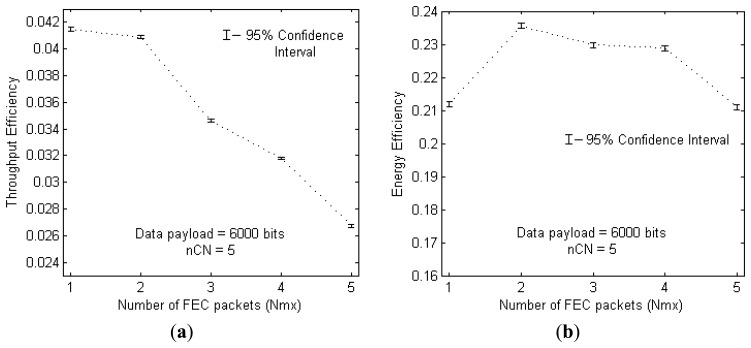
(**a**) Throughput efficiency *vs.* number of FEC packets (*N_mx_*); (**b**) Energy efficiency *vs. N_mx_*.

The effect of splitting the FEC part, into multiple FEC packets, on throughput efficiency and energy efficiency is shown in Figure 5a,b, respectively. For this analysis, the above achieved optimum payload size is used in a region with 5 *CNodes* distributed randomly at each run.

From Figure 5a, it is clear that throughput efficiency decreases as *N_mx_* is increased above 2. This is mainly because with more FEC fragments the total amount of time taken to transfer the required amount of FEC information to the destination increases; due to the accumulation of long propagation delays associated with each *NACK* and FEC packet transmission. The best throughputs are achieved at the values of *N_mx_* = 1 and 2, with *N_mx_* = 1 providing slightly better value. Thus it is clear that in the presence of long propagation delays it is better to transmit the FEC information in only one or two FEC packets than dividing it into many.

On the other hand, in terms of energy efficiency, the transmission of many smaller FEC packets does not rapidly degrade the performance (from Figure 5b). Transmission of smaller FEC packets, one-by-one, allow only up to the required amount of FEC information being sent to the destination, thus preventing the energy consumption from transmission of extra FEC bits. However, more number of FEC packets increases the total number of Viterbi decoding attempts, which take place every time a new packet is received, thus adding to the overall energy consumption. Because of these two opposing factors the energy efficiency does not show much deviation with an increase in *N_mx_*. However at *N_mx_* = 3 and *N_mx_* = 4, slight irregularities are, most probably, because of the puncturing patterns used during the simulations, but they are irrelevant to this analysis. The puncturing patterns used for generating FEC packets are shown in Table 2. The convolution code with the patterns used at *N_mx_* = 4 seems to perform equally better as that at *N_mx_* = 3, especially in terms of energy efficiency.

The 95% confidence interval shown at any point in Figure 5, let's say *N_mx_* = 1, is obtained over a total of 100 runs each delivering 1,000 packets. The average value of the throughput efficiency, obtained over 100 × 1,000 values, is found to be 0.0415 with a standard deviation of 0.0268. Then using the general principle of 95% confidence interval calculation, the one-sided interval can be approximated to 0.0002, thus resulting in the lower and upper 95% confidence levels of 0.0413 and 0.0417, respectively. Though not shown in other plots, the same confidence level applies for all other figures with number of runs varying from 100 to 150, and number of data packets varying from 1,000 to 1,500. Finally, utilising the results in Figure 5a and 5b simultaneously, we choose *N_mx_* = 2 to be the optimum value for number of FEC packets, whose energy efficiency is better than that of *N_mx_* = 1, with negligible difference in their throughput efficiencies.

### 5.3. Maximum Achievable Throughput Efficiency

**Figure 6.**
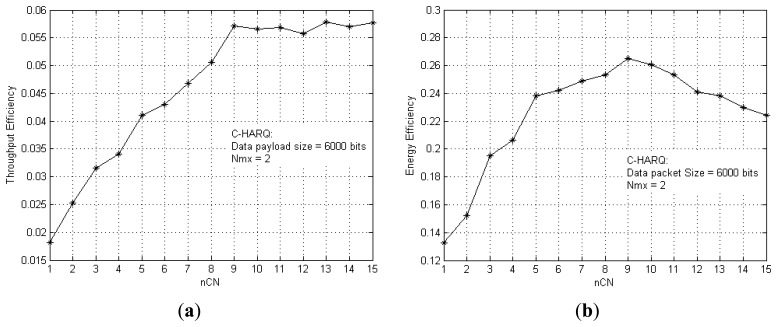
(**a**) Throughput efficiency *vs.* the number of nodes in the cooperative region (*nCN*); (**b**) Energy efficiency *vs. nCN*.

The improvement in throughput efficiency and energy efficiency due to an increase in the number of *CNodes* is shown in Figure 6a,b, respectively. More number of *CNodes* increases the probability of successfully delivering all the FEC packets to the destination, thus improving the performance. Figure 6a gives an idea of the minimum number of *CNodes* required to achieve the maximum achievable throughput efficiency, *i.e.*, *nCN* = 9. For *nCN* > 9, the extra *CNodes* are unable to enhance the throughput efficiency further. Rather, they decrease the energy efficiency by adding to the overall energy consumption of the network, as clearly depicted in Figure 6b. For very dense networks, these results can be utilised to set the limit for the *Look-Up* list length, defined earlier, so that once the optimum number of *CNodes* become available the *CNode* discovery process is skipped to directly go into cooperation, thus avoiding time and energy wastage.

### 5.4. Energy Consumption at Various Nodes

**Figure 7.**
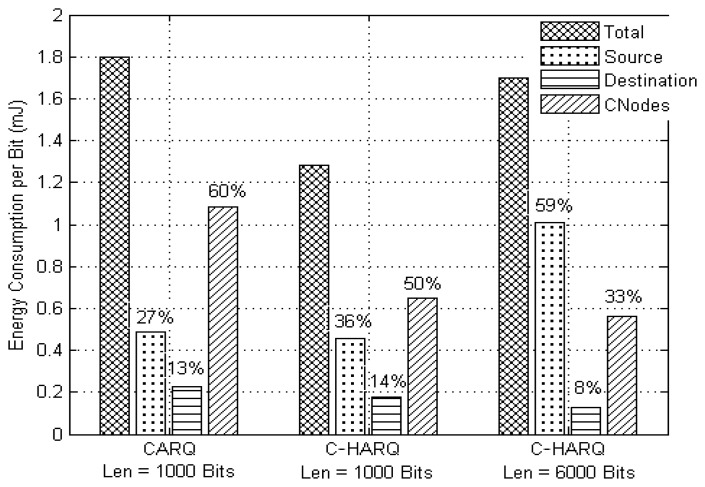
Average Energy consumption per bit (in mJ) at various nodes for CARQ and C-HARQ protocols, with *nCN* = 5.

In case of conventional S&W ARQ, it is obvious that most of the energy is consumed by the source node, compared to destination and *CNodes* (as *P_Tx_* > *P_Rx_* ≫ *P_Idle_*). In a long run, this method can quickly discharge a sensor node which is active most of the time, collecting and transferring data to a base station. In this regard, the cooperative protocols have the inherent advantage of distributing the energy consumption across the network by temporarily transferring the source's burden to the *CNodes*, thus allowing the source to conserve energy for future use. However, this approach of temporarily transferring the burden to *CNodes* by asking them to share their energy resources may cause a *CNode* to lose a significant amount of energy, thus affecting its own applications. The C-HARQ proves to be very efficient in this aspect as it asks the *CNodes* to carry the burden of delivering only the smaller FEC fragments while maintaining the source responsible for transmission of larger data packets. To illustrate this better, Figure 7 presents the energy consumption distribution in the network for CARQ and C-HARQ protocols, operating at their optimum settings. It gives an estimate of the average energy consumed per data bit by different nodes in successfully delivering the data from source to destination. The percentage value shown for a node denotes its percentage contribution to the total energy consumption. For the case of *CNodes*, the combined total energy consumption of all the *CNodes* is plotted (*nCN* = 5 in this case).

For the CARQ protocol, operating at its optimum throughput setting (*i.e.*, at payload length of 1,000 bits), the total energy consumed per data bit by all the *CNodes* is found to be greater than twice the energy consumed by the source; indicating that an individual *CNode* lose quite a significant amount of energy in providing cooperation or delivering other's data. On the other hand, C-HARQ (at 1,000 bits) not only consumes less energy but also reduces the burden of the *CNodes* by transferring some of the burden back to the source. When operating at its optimum payload length (6,000 bits), C-HARQ not only delivers a throughput efficiency of approximately four times the optimum throughput efficiency of CARQ (Figure 4a), at the expense of same or rather slightly less energy per bit, but also maintains the source responsible for major part of the energy consumption (by further transferring back the burden of *CNodes* to source). Thus, it can be said that C-HARQ provides an optimum performance between S&W ARQ and CARQ in terms of energy distribution. In other words, the use of error correction codes in C-HARQ helps a cooperative protocol to enhance its throughput while using the *CNodes* to deliver only the smaller FEC packets decreases the energy sharing burden of *CNodes*.

### 5.5. Performance at Varying Bit Error Rate (BER)

**Figure 8.**
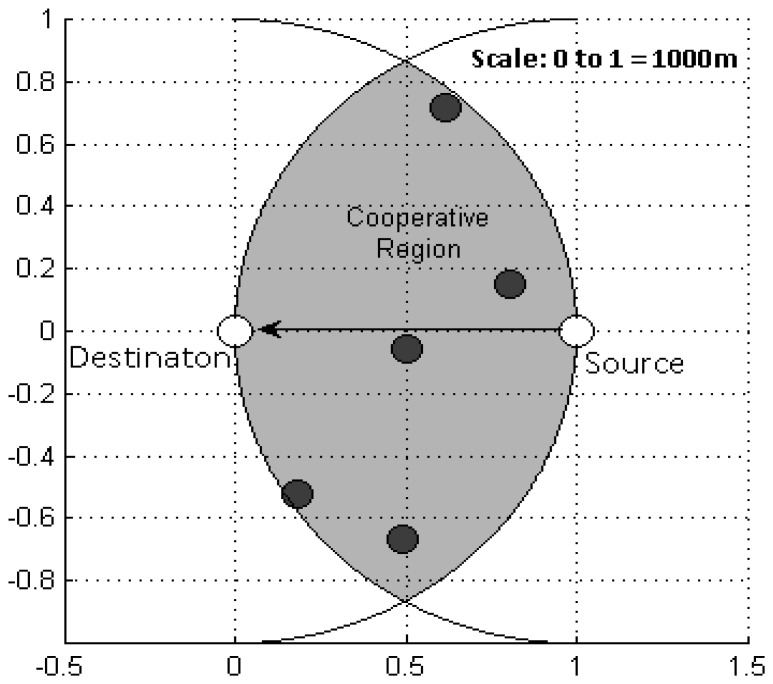
Network settings for BER analysis.

We first select a random network configuration by randomly distributing five cooperative nodes in the cooperative region, whose topography is shown in Figure 8.

The same as earlier, the BER of all the links in the network are obtained from the “average BER *vs.* the inter-nodal distance” graph [3]. Let's denote these obtained BER values of Source-to-Destination (S-D) link, *CNode*-to-Destination (CN-D) link and Source-to-*CNode* (S-CN) link with *ber_S-D*, *ber_CN(i)-D* and *ber_S-CN(i)*, respectively, where *i* ∈ {1,2,…*nCN*}These BER values are found to be of the order of 10^−3^. Now, to see the effect of BER variation of a particular link on the performance of C-HARQ, while the other links maintain their original BER values, we use a multiplication factor α such that it is multiplied to the original BER value of that particular link and varied over the required range. For example, the BER of the Source-to-Destination link can be varied, by varying the value of α in the relation: *BER_S-D* = α × *ber_S-D*.

#### 5.5.1. C-HARQ versus BER of Individual Links

**Figure 9.**
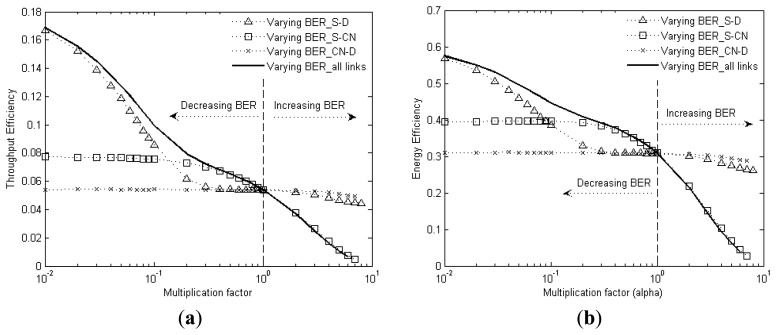
(**a**) Throughput efficiency and (**b**) Energy efficiency dependence on BER of different links, for C-HARQ, for data payload size = 6,000 bits, *Nmx* = 2 and *nCN* = 5.

Figure 9 shows the impact of BER variations of individual links on the throughput efficiency and energy efficiency. The BER of a link is individually varied by varying the value of α over the range shown in figure, while the others are kept constant at their original BER values. Additionally, the impact of an overall BER variation in the network is also obtained by multiplying α to all the links simultaneously.

The results reveal that the performance of C-HARQ depends mainly on the link conditions of S-D and S-CN channels. A decrease in *BER_S-D* causes a rapid performance improvement, however an increase in BER does not degrade the performance rapidly because of the error correction gains provided by convolution coding. The degradation in performance is mainly caused by the increase in *BER_S-CN* as these channels determine the availability of CNodes which can provide the required error correction information. Lastly, the CN-D link does not have any major impact because it is involved in only transferring the smaller FEC packets. Hence, it can be easily inferred that the performance of C-HARQ mainly depends on the links that are involved in transferring the data packet.

#### 5.5.2. C-HARQ, CARQ and S&W ARQ against BER of All Links

**Figure 10.**
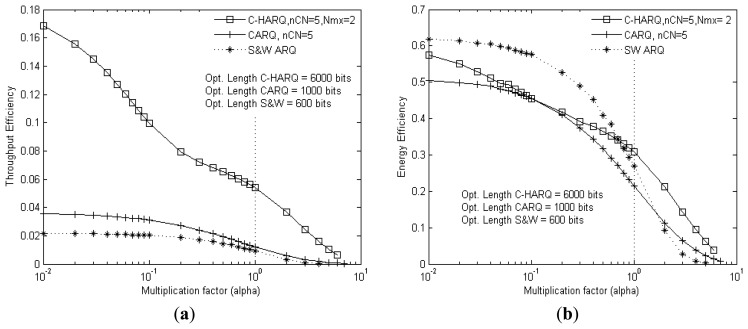
(**a**) Throughput efficiency; (**b**) Energy efficiency *vs.* BER of all links.

Figure 10a,b presents the throughput efficiency and energy efficiency comparison, respectively, against the BER variations of all the links at the same time.

Figure 10a clearly shows that C-HARQ outperforms CARQ and S&W, over the entire range of BER. However Figure 10b reveals that C-HARQ provides the best energy efficiency performance only at high BER, but at lower BER, S&W ARQ proves to be more energy efficient than the cooperative protocols. This is quite understandable, as under good channel conditions the cooperation process is not required so much and the cooperative protocols basically starts operating in S&W mode, but with the additional energy expenditure from *CNodes* due to overhearing of the original transmission. With five *CNodes* and ten times larger optimum payload size (compared to that of SW ARQ), these energy consumption becomes non negligible for C-HARQ and hence contributes to the degradation of the energy efficiency level.

## 6. Conclusions

Exploiting the efficiency of cooperative communication and the incremental error correction capabilities of the RCPC codes, an enhanced version of cooperative ARQ protocol is proposed in this paper for underwater acoustic sensor networks. This protocol efficiently merges the two ARQ schemes: Type-II HARQ and CARQ, so as to utilise the benefits of both the techniques. Computer simulations were done to show the enhanced performance of this hybrid cooperation technique by comparing its performance against the present Cooperative ARQ and standard S&W ARQ. The results show that not only is the throughput efficiency boosted to a much higher value, but the energy efficiency is also improved. Further, the optimum values of various network parameters were also estimated to extract the best performance from the proposed protocol. Using this basic framework of the C-HARQ protocol, more powerful rate compatible codes (e.g., rate compatible punctured turbo (RCPT) codes) can be employed to further improve the performance.

## Figures and Tables

**Table 1.** Simulation Parameters and Acoustic Modem Specifications.

**PARAMETER**	**VALUE**
Source-Destination distance	1,000 m
Sound Speed	1,500 m/s
*ACK/NACK* packet size	7 Bytes
*C_Req_*/*C_Avl_* packet size	7 Bytes
Data rate	20 kbps
Operating frequency	25 kHz
Transmission power (*P_Tx_*)	8 W
Reception power (*P_Rx_*)	0.8 W
Idle mode power (*P_Idle_*)	8 mW
Decoding energy per useful bit (*E_dec_*)	0.02 mJ

**Table 2.** Puncturing Patterns.

***No. of FEC Packets***	***FEC Packet***	***Puncturing Pattern Used for Generating the Related FEC Packet***
*N_mx_* = 0	*Pac*-0	10101010101010101010

*N_mx_* = 1	*Pac*-1	01010101010101010101

*N_mx_* = 2	*Pac*-1	01000100010001000100
	*Pac*-2	00010001000100010001

*N_mx_* = 3	*Pac*-1	01000001000001000001
	*Pac*-2	00010000010000010000
	*Pac*-3	00000100000100000100

*N_mx_* = 4	*Pac*-1	01000000010000000100
	*Pac*-2	00010000000100000001
	*Pac*-3	00000100000001000000
	*Pac*-4	00000001000000010000

*N_mx_* = 5	*Pac*-1	01000000000100000000
	*Pac*-2	00010000000001000000
	*Pac*-3	00000100000000010000
	*Pac*-4	00000001000000000100
	*Pac*-5	00000000010000000001
